# New Publications

**Published:** 1895-06

**Authors:** 


### NEW PUBLICATIONS.

In the domain of veterinary science so much good material has recently been added that we can only briefly allude to some of the most important and valuable additions with which the profession has been favored the past two years.

Looking over the field of veterinary medicine we find added to our storehouse the recent edition of Hoare's *Manual of Veterinary Therapeutics and Pharmacology*, which brings to us fresh knowledge gained through many sources and presents for our pleasure its contents in a new and attractive form.

A fifth edition of Tuson's *Veterinary Pharmacopœia* has recently been issued, which is the best evidence of the stronghold this book still retains among the profession. The ready manner with which one can refer to the actions and uses, preparations, incompatibles, etc., of each drug still insures for it a true place in our libraries and pharmacies.

With these might be mentioned the *Veterinary Pharmacopœia, Materia Medica, and Therapeutics* of the Doctors Gresswell, with clear descriptions of and the physiological actions of medicines to enable one to become more thoroughly posted in this important branch of our every-day work.

In the direction of surgery, where so much grand work has been accomplished the past few years and which has been so much simplified to us, by a greater knowledge of anæsthesia, general and local, newer modes of restraint, operating tables, etc., we have had much new light shed on such important operations as laryngotomy, through the work of Prof. Fleming and through the translation by Prof. Dollar of the French work of Prof. Cadiot.

In addition to these we have had that valuable contribution of that eminent veterinary surgeon, Prof. A. Liautard, whose work comes to us none too soon, for our needs were great.

Along with these free-will offerings, we are now promised this year the second volume of Fleming's *Surgery*. We are quite sure that our every reader will be glad to learn this news from our columns, for all who have been favored by the possession of the first volume prize it highly for its clearness and completeness.

Later news comes to us from that ever-progressive veterinary publishing house of New York City, W. R. Jenkins, that they have in preparation a translation of Mallir's *Surgery*, all of which will be gladly learned by every veterinarian.

In another direction we have had a special contribution for our study and consideration in the book prepared by Dr. David Roberge on *Lameness and Diseases of the Foot Traced to an Unbalanced Foot-bone, and the Value of Balancing the Foot.*

Of the publication in English, by Prof. W. L. Zuill, of Friedberger & Frohner's *Pathology and Therapeutics of the Domestic Animals*, it can be well said that it fills in great measure a long-felt want and will be read and re-read with increasing pleasure and a better understanding of this branch of our work day by day.

Drs. J. B. & A. Gresswell have added the second edition, enlarged, of their *Manual of the Theory and Practice of Equine Medicine.*

To these have been added, through the untiring labors of Prof. Fleming, to whom we are all so greatly indebted, the translation of that comprehensive work of Prof. L. G. Neumann, of Toulouse, concerning *Parasites and Parasitic Diseases of Domesticated Animals*, a subject of great importance and given entirely too little consideration at the hands of the general practitioner.

*Horses, in Accident and Disease*, has been added for general reading and for students by Mr. J. Roalfe Cox, Fellow of the Royal College Veterinary Surgeons.

A more recent work partially devoted to the *Diseases of Cats* has been added to our literature by Prof. R. S. Huidekoper, and well merits the attention of city veterinarians under whose care these members of the feline family frequently fall.

*The Laws of Sale and Warranty* and the all-important question of *Soundness or Unsoundness in Horses*, a subject that greets us daily and tries our very souls, has been prepared for our better guidance by Mr. J. Irwin Lupton, Fellow of the Royal College of Veterinary Surgeons. Works of this character are ever sought out by the veterinarians of large cities, where this work of inspection forms a large part of the daily routine.

The ever-interesting field of physiology with all its important relations in our sphere of work has not been forgotten in the literary progress of the near past, and this boon, emanating from the pen of Veterinary Capt. F. Smith, M.R.C.V.S., makes a very distinctive claim in that it is a strictly veterinary physiology.

From the same author has also emanated a second edition of a closely allied work, a book destined to stand for us as a *Manual of Veterinary Hygieue.* The importance of this subject, so much more fully realized and appreciated since the subject of bovine tuberculosis has been uppermost in our thoughts and plans, makes it a valuable line of thought, and will no doubt lead to other works in this direction and be a means of establishing for the domesticated animals a better condition of affairs, a higher standard of stable hygiene, and better sanitary environments.

Microbiology, a subject of so much importance in the present status of medicine, is concisely considered by Profs. Mosselman and Leinaux, of the National Veterinary College of Cureghem, Belgium, in their work so well received abroad. Through the labors of Prof. R. R. Dinwiddie this work has been translated into English, and will be read by every progressive veterinarian in America and Great Britain.

*The Book on the Cat*, by Prof. R. S. Huidekoper, published by D. Appleton & Co., has reached our tables, and it will well fill for a time a serious void in our libraries as a guide to the classification and varieties of cats, and add to our knowledge of their care, diseases, and treatment. Its greatest fault is its brevity, and the numerous ailments affecting the members of the feline family are only treated upon in a more or less general manner. We trust that it will prove a fruitful source of interest and that its reception will lead to a more thorough and exhaustive treatise in this sphere of our work.

To these very valuable works have been added many others, treating of specific subjects, lines of investigation, newer methods of horseshoeing, etc., but we have briefly called attention to a few of the most important, and it will come as a rude awakening to many who have not summed up from time to time the great and good growth of English veterinary literature.

